# Editorial: Lineage tracing, hematopoietic stem cell and immune cell dynamics

**DOI:** 10.3389/fimmu.2022.1062415

**Published:** 2022-10-18

**Authors:** Caleb A. Lareau, Chiara Romagnani, Leif S. Ludwig

**Affiliations:** ^1^ Department of Pathology, Stanford University, Stanford, CA, United States; ^2^ Innate Immunity, Deutsches Rheuma-Forschungszentrum Berlin (DRFZ), ein Leibniz Institut, Berlin, Germany; ^3^ Medical Department I, Charité University Medicine Berlin, Berlin, Germany; ^4^ Leibniz-Science Campus Chronic Inflammation, Berlin, Germany; ^5^ Berlin Institute of Health at Charité Universitätsmedizin Berlin, Berlin, Germany; ^6^ Max‐Delbrück‐Center (MDC) for Molecular Medicine in the Helmholtz Association, Berlin Institute for Medical Systems Biology (BIMSB), Berlin, Germany

**Keywords:** lineage tracing, hematopoietic stem cells, clonal dynamics, single cell multi-omics, immunology, cancer biology

Lineage tracing has enabled intimate insights into cellular relationships and dynamics of developing blood and immune cells, their homeostasis and regeneration, and dysregulation in disease (1). By revealing where and when cells originate and following the fate of (individual) cells, lineage tracing has advanced our understanding of differentiation, cell fate, malignant transformation, and adaptive responses to environmental cues. In light of advances in single cell multi-omics, we summarize new directions enabled by works from this Research Topic.

Penter et al. review tools for the high-resolution monitoring of disease evolution, focusing on naturally occurring genetic alterations for *in vivo* lineage tracing in human clinical specimens. For example, immune cell receptor V(D)J rearrangements of T cell and B cell receptors have long served to investigate clonal dynamics, heterogeneity, and responses of the immune system in disease settings. To study clonal dynamics and phylogenetically dissect subclones in malignancies, chromosomal copy number variants have been invaluable (2), given their relative ease of detection *via* single cell sequencing. By contrast, the *de novo* detection of single nucleotide variants in the nuclear genome of individual cells is more challenging. Still, these variants are often directly linked to altered gene functions that play major roles in tumorigenesis, making their co-detection highly desirable (3). More recently, the utility of somatic mitochondrial DNA (mtDNA) mutations has been recognized to study clonal dynamics and evolution (4). Given the distinct genetic material of mitochondria, approaches to capture natural mtDNA genetic variation are readily compatible with co-detecting somatic nuclear variants, as well as integrating cell state measurements *via* single cell RNA- and ATAC-seq. The co-detection of cell state and somatic variation will be an essential pairing for future technologies. The authors further discuss the role of these approaches to tackle open questions in hematopoietic stem cell (HSC) transplantation.

Konturek-Ciesla and Bryder provide a review of methodologies and perspectives of HSC lineage tracing in mice and humans. While hematopoiesis may represent one of the best-studied biological systems that has served as a paradigm for stem cell biology (5), recent technical advances continue to provide novel insights into HSC biology. The authors reflect on the foundational role of transplantation experiments to assay HSC function and how this informed HSC heterogeneity, lineage bias, and evolution over development and age. They discuss the refinement of cellular barcoding *via* (viraly) introducing heritable DNA tags and how the combination with mRNA expression profiling *via* single cell genomics has facilitated molecular insights into the regulatory circuits that orchestrate HSC fate and function. Further, the authors note key biological differences between HSC transplantation and native hematopoiesis, highlighting genetic models that leverage variations of Cre recombinase and CRISPR systems. These approaches enable temporal control of HSC labeling and investigate HSC biology with molecular profiling in more physiologic contexts than transplantation and *in vitro* assays. Last, they discuss advances and the challenge of lineage tracing in humans using somatic nuclear and mtDNA mutations and the utility of viral integration sites to study hematopoiesis in gene therapy patients (6).

DePasquale et al. highlight the utility of single cell genomics to investigate the interplay of blood malignancies and the immune system. They apply single-cell RNA-seq to dissect cellular heterogeneity in blastic plasmacytoid dendritic cell neoplasm (BPDCN) and the associated immune microenvironment. As plasmacytoid dendritic cells are key secreters of type I interferons (e.g., interferon A/INFA) which can modulate immunotherapy outcomes, the study of their transformed counterparts provides a unique context to study interferon dysregulation in cancer immunity. By profiling the bone marrow of BPDCN patients the authors revealed an altered balance between INFA and TNF signaling in T cells, which are likely to impact immune function. By linking the T cell receptor repertoire to transcriptomics profiles, the authors further characterize CD8^+^ memory T cell exhaustion in this disease context. This work provides a clear motivation to further elucidate the interplay between cancer and immune cells which will be essential to identify effective treatments leveraging tumor-reactive T cells.

New treatment avenues are also the focus in a perspective by Gutierrez et al. Over the years, a variety of models of cancer evolution have been introduced to account for the increasingly appreciated genetic and non-genetic heterogeneity of tumors that contribute to therapy resistance. The authors discuss the shortcomings of classical chemotherapy and targeted approaches as well as cancer diagnostics. Importantly, in many instances, current treatments cannot outpace the full evolutionary potential of cancers. To overcome these limitations, they discuss the potential of homogenization therapy and how functionalized lineage tracing *via* integrated multi-omic phenotyping is a means to measure this evolution. In essence, homogenization attempts to drive all tumor cells (regardless of genetic background or disparate cellular states) to a common targetable phenotype. The authors discuss evidence that supports homogenization and outline open questions and challenges, including investigating tumors in their spatial context, along this road towards proactive (rather than reactive approaches to) cancer therapy.

The importance of spatial context and inter- and multi-cellular communication in orchestrating cell function and fate is further highlighted in a perspective by Albers and Pelka. The authors introduce the concept of multicellular hubs, which comprise five key features: 1) they are spatially organized, with the function being defined by their composition, activity, and location. 2) The function is thereby the result of cellular cooperation, with each cell state/type fulfilling a specific task. 3) The resulting interactions may alter the cell state and developmental trajectory of its components and descending cells. 4) As such, multicellular hubs are dynamic and readily respond to environmental cues. 5) Similar to cells, multicellular hubs may therefore exist in different activity states. The authors review avenues of profiling multicellular hubs in primary human tissues, outlining challenges and open questions to characterize their functional role.

Together, this Research Topic synthesizes the advances in the field, outlining important avenues forward to study cellular dynamics. Current and future approaches that expand the characterization and interplay of cells and organizational structures will further identify key regulators of physiologic processes in human tissues.

## Author contributions

All authors contributed equally to shaping the Research Topic and the editorial.

## Acknowledgments

The authors want to thank all authors for their contributions and the staff of Frontiers for their support to enable this Research Topic.

## Conflict of interest

LL and CL are named on patents and applications related to technologies to enable mitochondrial DNA genotyping in single cells.

The remaining author declares that the research was conducted in the absence of any commercial or financial relationships that could be construed as a potential conflict of interest.

## Publisher’s note

All claims expressed in this article are solely those of the authors and do not necessarily represent those of their affiliated organizations, or those of the publisher, the editors and the reviewers. Any product that may be evaluated in this article, or claim that may be made by its manufacturer, is not guaranteed or endorsed by the publisher.
